# F179. NEURAL CORRELATES IN MUSICAL DEFICITS IN SCHIZOPHRENIA

**DOI:** 10.1093/schbul/sby017.710

**Published:** 2018-04-01

**Authors:** Ken Sawada, Sanae Hatada, Ryoshuke Fujito, William Honer

**Affiliations:** 1 Kochi Medical School; 2 Tosa Rehabilitation College; 3 Fujito Hospital; 4 University of British Columbia

## Abstract

**Background:**

Several studies have shown that patients with schizophrenia have low musical ability that correlates with poor cognitive functions and severe negative symptoms. Recently, using surface-based analysis, we reported that thinner cortical thickness in the left temporal, parietal, and inferior frontal regions is associated with lower musical ability in schizophrenia. Interestingly, thicker cortical thickness in the left supramarginal region correlates with lower musical ability in controls. Musical deficits such as congenital amusia was shown to contribute to white and grey matter pathophysiology in schizophrenia. We, therefore, sought to investigate diffusion tensor images (DTI) of patients with schizophrenia using an automated probabilistic tractography algorithm.

**Methods:**

Twenty-two right-handed patients with schizophrenia (12 males and 10 females, mean age = 45.9 years) and 20 right-handed healthy control subjects (13 males and 7 females, mean age = 42.8 years) consented to participate in this study. We measured musical ability, cognitive functions, and clinical assessments using the Montreal Battery for Evaluation of Amusia (MBEA), Brief Assessment of Cognition in Schizophrenia (BACS), and Positive and Negative Syndrome Scale (PANSS), respectively. We employed automatic probabilistic tractography DTI analysis using TRActs Constrained by UnderLying Anatomy (TRACULA) available in the Freesurfer software for the reconstruction of major tract bundles.

**Results:**

Whole-tract diffusion characteristics in patients with schizophrenia and controls were significantly different. Fractional anisotropy (FA) was lower for patients with schizophrenia compared to controls in the left superior longitudinal fasciculus - parietal endings (slfp) (p < 0.001), left cingulum - angular bundle (cab) (p < 0.001), and corpus callosum - forceps minor (fminor) (p < 0.001). We found significant correlation between musical abilities and FA alterations in slfp in both controls and patients with schizophrenia. While lower musical ability corresponds to lower FA in slfp of controls (r = -0.572, p = 0.013), it is associated with higher FA in the slfp of patients with schizophrenia (r = 0.515, p = 0.021).

**Discussion:**

This study shows that TRACULA can be used for the detection of decrements in several DTI tracts including the left slfp, left cab, and fminor in patients with schizophrenia. It revealed that while lower musical ability correlates with lower FA values in the left slfp in controls, it is associated with higher FA values in the same region in patients with schizophrenia. This contradictory finding in controls and patients with schizophrenia with regard to white matter pathology may reflect left supramarginal region malfunction resulting in cortical pathology in patients with schizophrenia. The data suggest that patients with schizophrenia may be more susceptible to changes in cortical thickness in the supramarginal region, and white matter alteration in the left slfp. Further study is needed to confirm the results. The characteristics of grey and white matter in the left parietal region which are relevant to musical ability may provide insight into pathological progression in patients with schizophrenia.

